# Supplementary material for: Population-scale analysis of common and rare genetic variation associated with hearing loss in adults
Source: Commun Biol. 2022 Jun 3;5:540. doi: 10.1038/s42003-022-03408-7 (PMC9166757; doi:10.1038/s42003-022-03408-7)
Supplement: Supplementary file 2 — Supplementary Information [file 42003_2022_3408_MOESM2_ESM.pdf]

SUPPLEMENTARY INFORMATION.
Praveen et al. 2022

Table of Contents

SUPPLEMENTARY INFORMATION GUIDE....2

SUPPLEMENTARY FIGURES....3

SUPPLEMENTARY FIGURE 1....3
SUPPLEMENTARY FIGURE 2....4
SUPPLEMENTARY FIGURE 3....13
SUPPLEMENTARY FIGURE 4....14

SUPPLEMENTARY METHODS....15

Supplementary Information Guide

Supplementary Information:

- Supplementary figures 1-4
- Supplementary Methods

Supplementary Data:

- Supplementary data 1-11

SUPPLEMENTARY FIGURES

Supplementary Figure 1.

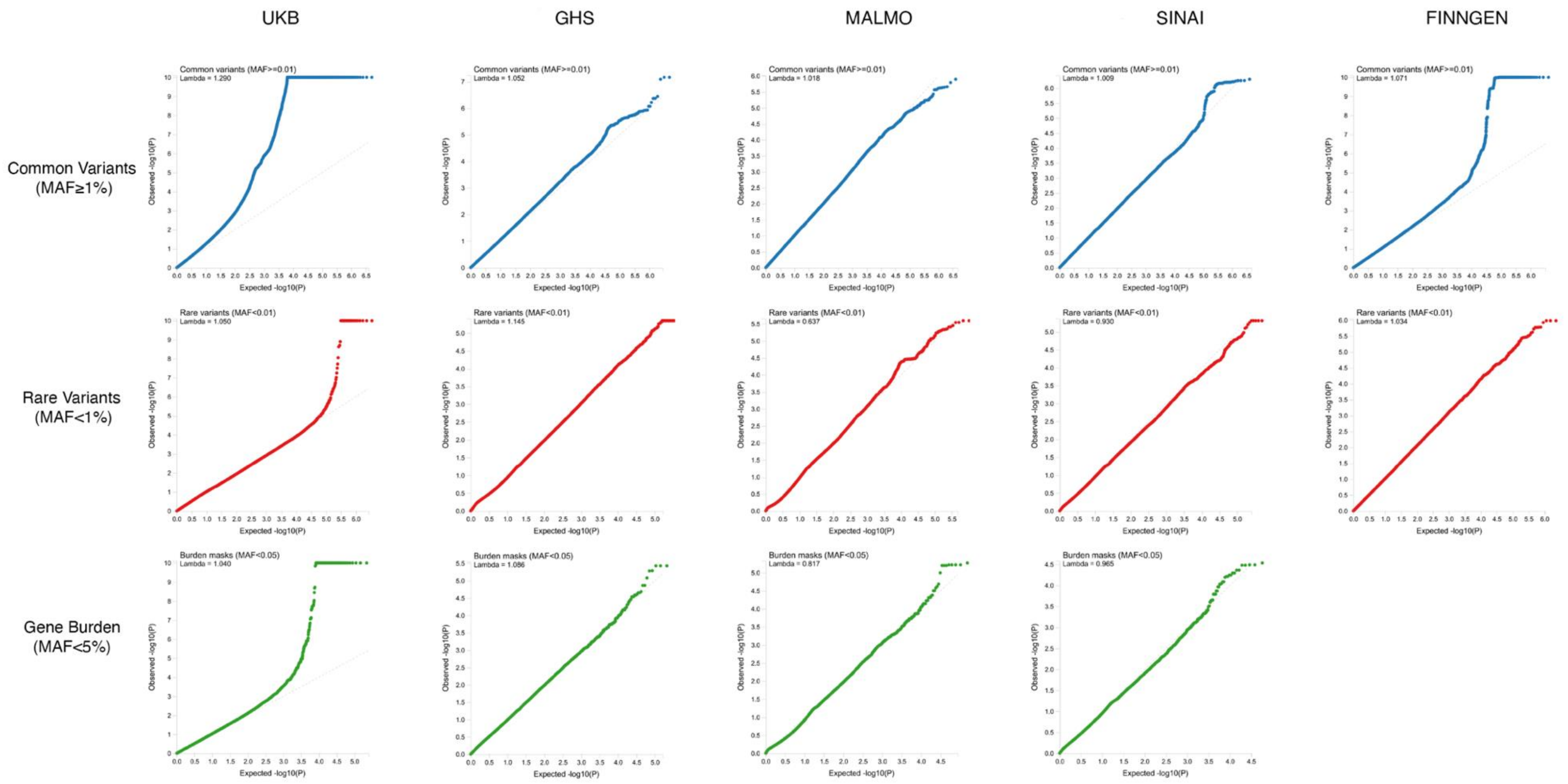

Supplementary Figure 1: Q-Q plots for common and rare single variant and gene burden associations in UKB, GHS, MALMO, SINAI and FinnGen.

Supplementary Figure 2.

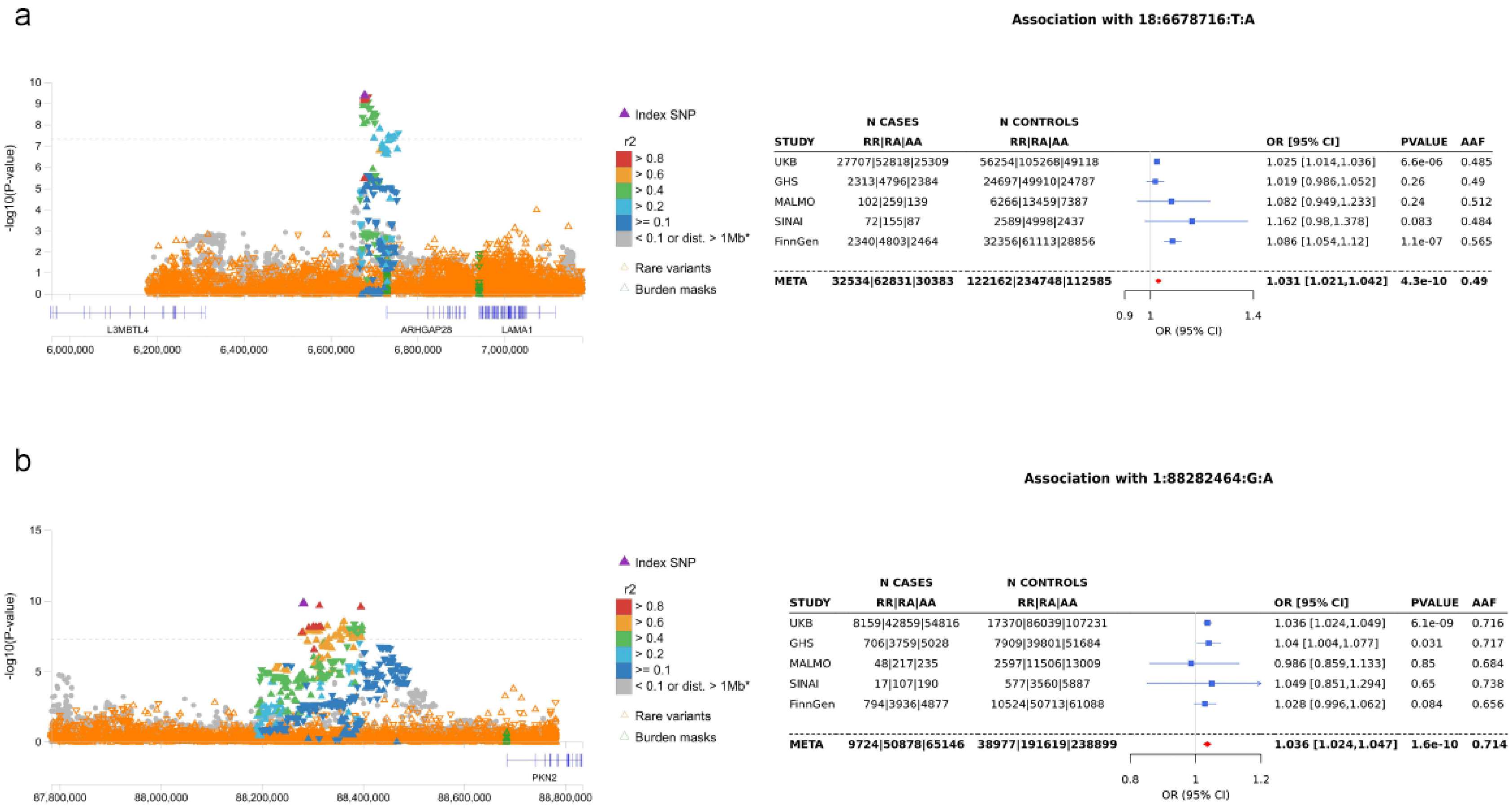

c

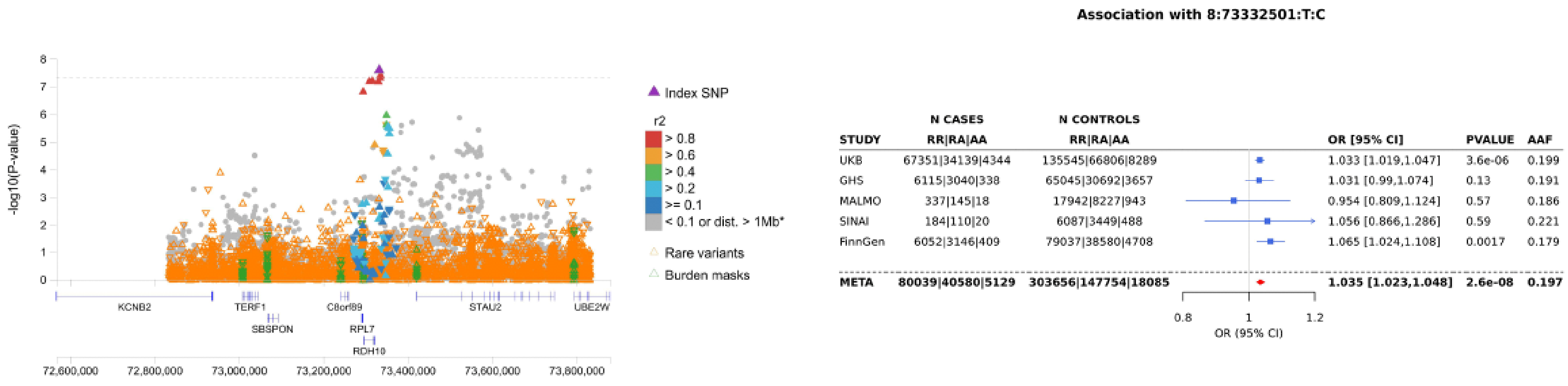

-log10(P-value)
Index SNP
r2
> 0.8
> 0.6
> 0.4
> 0.2
>= 0.1
< 0.1 or dist. > 1Mb*
Rare variants
Burden masks
KCNB2
TERF1
SBSPON
C8orf89
RPL7
RDH10
STAU2
UBE2W
72,600,000
72,800,000
73,000,000
73,200,000
73,400,000
73,600,000
73,800,000
Association with 8:73332501:T:C
STUDY | N CASES RR|RA|AA | N CONTROLS RR|RA|AA | OR [95% CI] | PVALUE | AAF
UKB | 67351|34139|4344 | 135545|66806|8289 | 1.033 [1.019,1.047] | 3.6e-06 | 0.199
GHS | 6115|3040|338 | 65045|30692|3657 | 1.031 [0.99,1.074] | 0.13 | 0.191
MALMO | 337|145|18 | 17942|8227|943 | 0.954 [0.809,1.124] | 0.57 | 0.186
SINAI | 184|110|20 | 6087|3449|488 | 1.056 [0.866,1.286] | 0.59 | 0.221
FinnGen | 6052|3146|409 | 79037|38580|4708 | 1.065 [1.024,1.108] | 0.0017 | 0.179
META | 80039|40580|5129 | 303656|147754|18085 | 1.035 [1.023,1.048] | 2.6e-08 | 0.197
0.8
1
1.2
OR (95% CI)

d

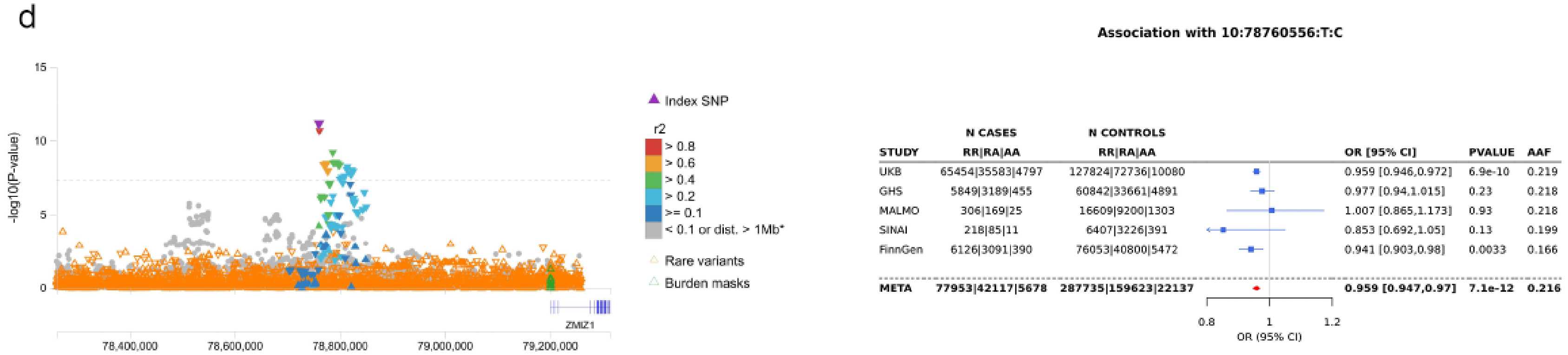

-log10(P-value)
Index SNP
r2
> 0.8
> 0.6
> 0.4
> 0.2
>= 0.1
< 0.1 or dist. > 1Mb*
Rare variants
Burden masks
ZMIZ1
78,400,000
78,600,000
78,800,000
79,000,000
79,200,000
Association with 10:78760556:T:C
STUDY | N CASES RR|RA|AA | N CONTROLS RR|RA|AA | OR [95% CI] | PVALUE | AAF
UKB | 65454|35583|4797 | 127824|72736|10080 | 0.959 [0.946,0.972] | 6.9e-10 | 0.219
GHS | 5849|3189|455 | 60842|33661|4891 | 0.977 [0.94,1.015] | 0.23 | 0.218
MALMO | 306|169|25 | 16609|9200|1303 | 1.007 [0.865,1.173] | 0.93 | 0.218
SINAI | 218|85|11 | 6407|3226|391 | 0.853 [0.692,1.05] | 0.13 | 0.199
FinnGen | 6126|3091|390 | 76053|40800|5472 | 0.941 [0.903,0.98] | 0.0033 | 0.166
META | 77953|42117|5678 | 287735|159623|22137 | 0.959 [0.947,0.97] | 7.1e-12 | 0.216
0.8
1
1.2
OR (95% CI)

e

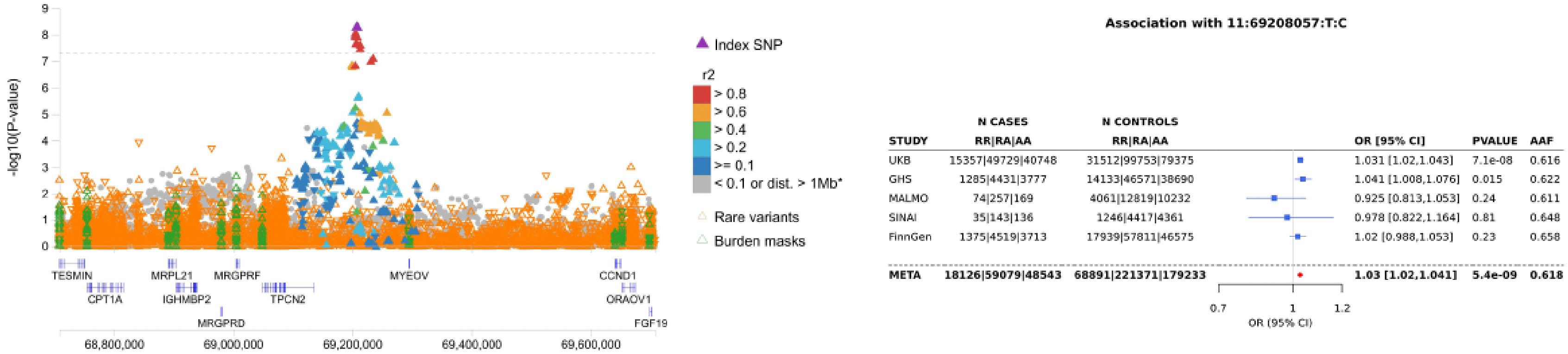
Index SNP
r2
> 0.8
> 0.6
> 0.4
> 0.2
>= 0.1
< 0.1 or dist. > 1Mb*
Rare variants
Burden masks
-log10(P-value)
TESMIN
MRPL21
MRGPRF
MYEOV
CCND1
CPT1A
IGHMBP2
TPCN2
ORAOV1
MRGPRD
FGF19
68,800,000
69,000,000
69,200,000
69,400,000
69,600,000
Association with 11:69208057:T:C
STUDY
N CASES RR|RA|AA
N CONTROLS RR|RA|AA
OR [95% CI]
PVALUE
AAF
UKB 15357|49729|40748 31512|99753|79375 1.031 [1.02,1.043] 7.1e-08 0.616
GHS 1285|4431|3777 14133|46571|38690 1.041 [1.008,1.076] 0.015 0.622
MALMO 74|257|169 4061|12819|10232 0.925 [0.813,1.053] 0.24 0.611
SINAI 35|143|136 1246|4417|4361 0.978 [0.822,1.164] 0.81 0.648
FinnGen 1375|4519|3713 17939|57811|46575 1.02 [0.988,1.053] 0.23 0.658
META 18126|59079|48543 68891|221371|179233 1.03 [1.02,1.041] 5.4e-09 0.618
0.7
1
1.2
OR (95% CI)

f

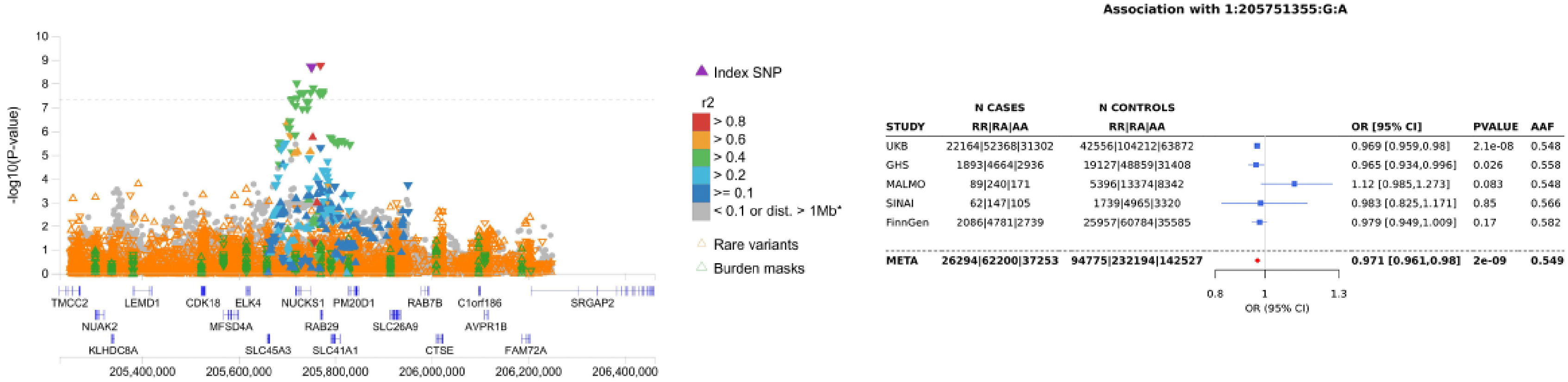
Index SNP
r2
> 0.8
> 0.6
> 0.4
> 0.2
>= 0.1
< 0.1 or dist. > 1Mb*
Rare variants
Burden masks
-log10(P-value)
TMCC2
LEMD1
CDK18
ELK4
NUCKS1
PM20D1
RAB7B
C1orf186
SRGAP2
NUAK2
MFSD4A
RAB29
SLC26A9
AVPR1B
KLHDC8A
SLC45A3
SLC41A1
CTSE
FAM72A
205,400,000
205,600,000
205,800,000
206,000,000
206,200,000
206,400,000
Association with 1:205751355:G:A
STUDY
N CASES RR|RA|AA
N CONTROLS RR|RA|AA
OR [95% CI]
PVALUE
AAF
UKB 22164|52368|31302 42556|104212|63872 0.969 [0.959,0.98] 2.1e-08 0.548
GHS 1893|4664|2936 19127|48859|31408 0.965 [0.934,0.996] 0.026 0.558
MALMO 89|240|171 5396|13374|8342 1.12 [0.985,1.273] 0.083 0.548
SINAI 62|147|105 1739|4965|3320 0.983 [0.825,1.171] 0.85 0.566
FinnGen 2086|4781|2739 25957|60784|35585 0.979 [0.949,1.009] 0.17 0.582
META 26294|62200|37253 94775|232194|142527 0.971 [0.961,0.98] 2e-09 0.549
0.8
1
1.3
OR (95% CI)

g

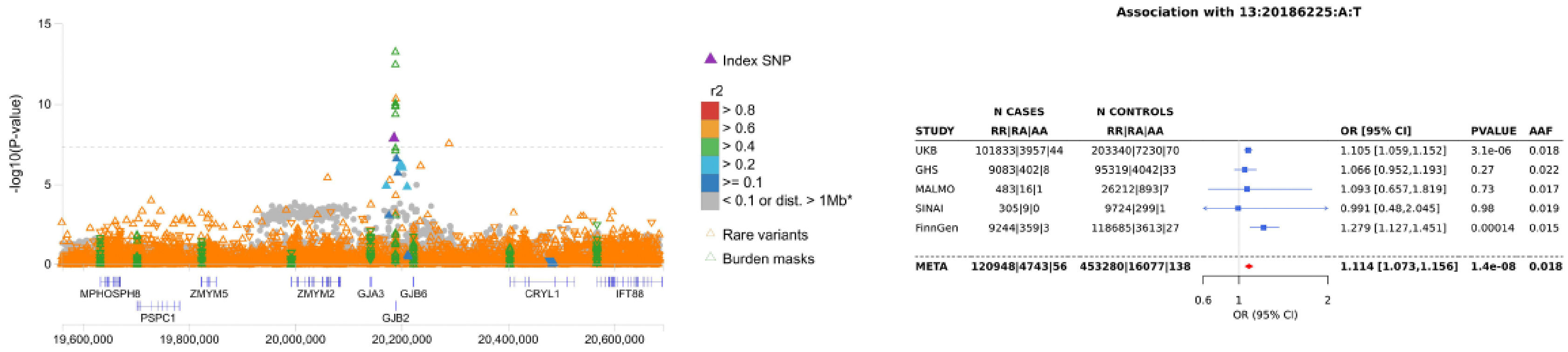
-log10(P-value)
15
10
5
0
MPHOSPH8
ZMYM5
ZMYM2
GJA3
GJB6
CRYL1
IFT88
PSPC1
GJB2
19,600,000
19,800,000
20,000,000
20,200,000
20,400,000
20,600,000
Index SNP
r2
> 0.8
> 0.6
> 0.4
> 0.2
>= 0.1
< 0.1 or dist. > 1Mb*
Rare variants
Burden masks
Association with 13:20186225:A:T
STUDY | N CASES RR|RA|AA | N CONTROLS RR|RA|AA | OR [95% CI] | PVALUE | AAF
UKB | 101833|3957|44 | 203340|7230|70 | 1.105 [1.059,1.152] | 3.1e-06 | 0.018
GHS | 9083|402|8 | 95319|4042|33 | 1.066 [0.952,1.193] | 0.27 | 0.022
MALMO | 483|16|1 | 26212|893|7 | 1.093 [0.657,1.819] | 0.73 | 0.017
SINAI | 305|9|0 | 9724|299|1 | 0.991 [0.48,2.045] | 0.98 | 0.019
FinnGen | 9244|359|3 | 118685|3613|27 | 1.279 [1.127,1.451] | 0.00014 | 0.015
META | 120948|4743|56 | 453280|16077|138 | 1.114 [1.073,1.156] | 1.4e-08 | 0.018
0.6
1
2
OR (95% CI)

h

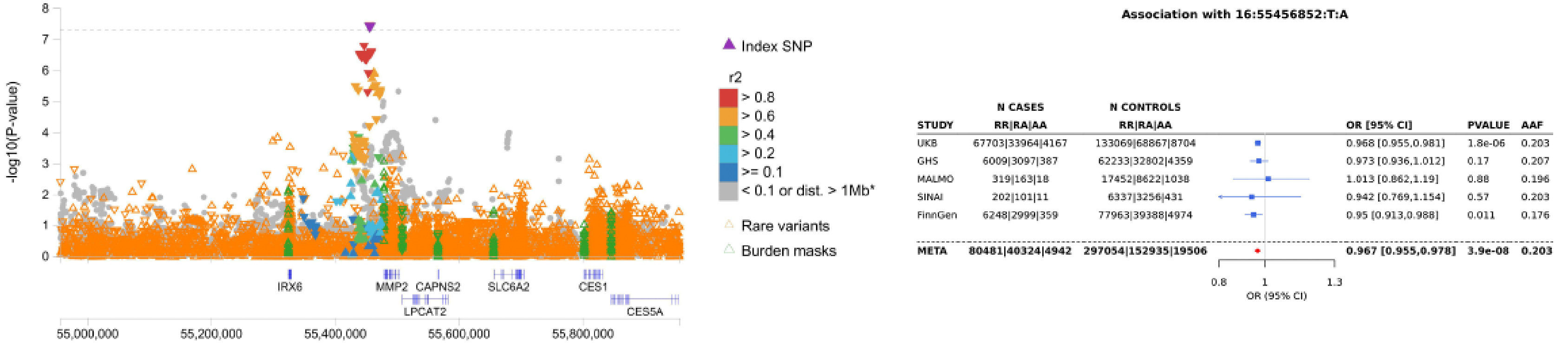
-log10(P-value)
8
7
6
5
4
3
2
1
0
IRX6
MMP2
CAPNS2
SLC6A2
CES1
LPCAT2
CES5A
55,000,000
55,200,000
55,400,000
55,600,000
55,800,000
Index SNP
r2
> 0.8
> 0.6
> 0.4
> 0.2
>= 0.1
< 0.1 or dist. > 1Mb*
Rare variants
Burden masks
Association with 16:55456852:T:A
STUDY | N CASES RR|RA|AA | N CONTROLS RR|RA|AA | OR [95% CI] | PVALUE | AAF
UKB | 67703|33964|4167 | 133069|68867|8704 | 0.968 [0.955,0.981] | 1.8e-06 | 0.203
GHS | 6009|3097|387 | 62233|32802|4359 | 0.973 [0.936,1.012] | 0.17 | 0.207
MALMO | 319|163|18 | 17452|8622|1038 | 1.013 [0.862,1.19] | 0.88 | 0.196
SINAI | 202|101|11 | 6337|3256|431 | 0.942 [0.769,1.154] | 0.57 | 0.203
FinnGen | 6248|2999|359 | 77963|39388|4974 | 0.95 [0.913,0.988] | 0.011 | 0.176
META | 80481|40324|4942 | 297054|152935|19506 | 0.967 [0.955,0.978] | 3.9e-08 | 0.203
0.8
1
1.3
OR (95% CI)

i

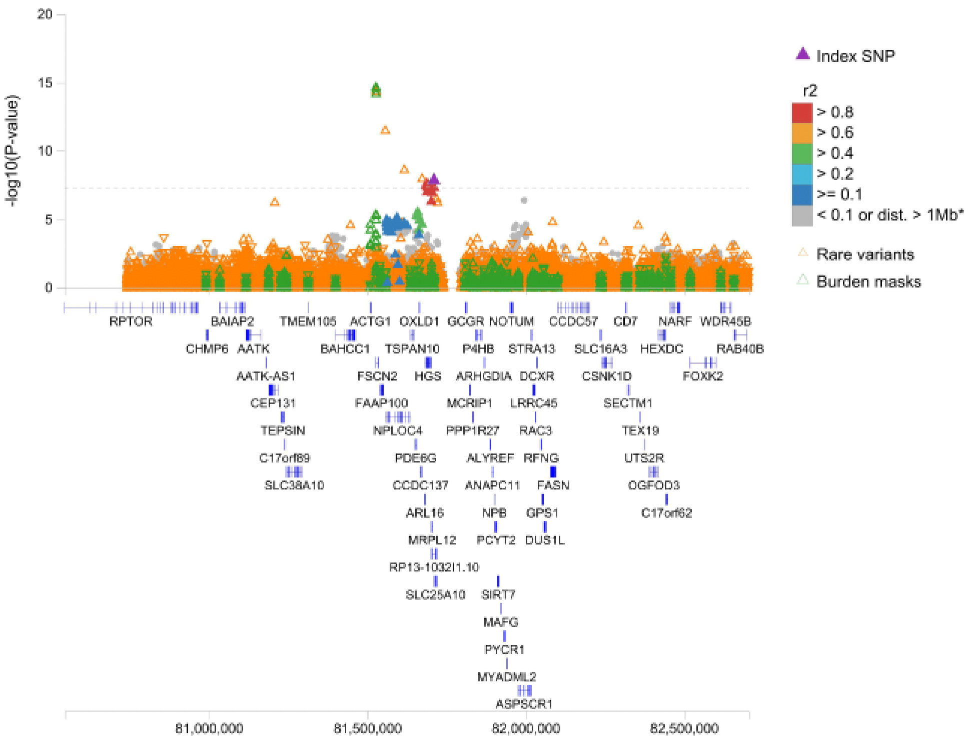
-log10(P-value)
20
15
10
5
0
Index SNP
r2
> 0.8
> 0.6
> 0.4
> 0.2
>= 0.1
< 0.1 or dist. > 1Mb*
Rare variants
Burden masks
RPTOR
BAIAP2
TMEM105
ACTG1
OXLD1
GCGR
NOTUM
CCDC57
CD7
NARF
WDR45B
CHMP6
AATK
BAHCC1
TSPAN10
P4HB
STRA13
SLC16A3
HEXDC
RAB40B
AATK-AS1
FSCN2
HGS
ARHGDIA
DCXR
CSNK1D
FOXK2
CEP131
FAAP100
MCRIP1
LRRC45
SECTM1
TEPSIN
NPLOC4
PPP1R27
RAC3
TEX19
C17orf89
PDE6G
ALYREF
RFNG
UTS2R
SLC38A10
CCDC137
ANAPC11
FASN
OGFOD3
ARL16
NPB
GPS1
C17orf62
MRPL12
PCYT2
DUS1L
RP13-1032I1.10
SLC25A10
SIRT7
MAFG
PYCR1
MYADML2
ASPSCR1
81,000,000
81,500,000
82,000,000
82,500,000

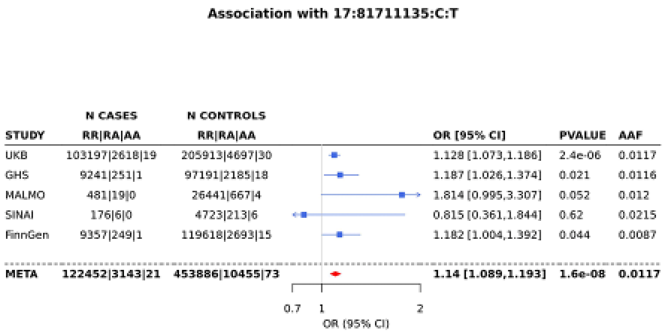
Association with 17:81711135:C:T
STUDY
N CASES RR|RA|AA
N CONTROLS RR|RA|AA
OR [95% CI]
PVALUE
AAF
UKB 103197|2618|19 205913|4697|30 1.128 [1.073,1.186] 2.4e-06 0.0117
GHS 9241|251|1 97191|2185|18 1.187 [1.026,1.374] 0.021 0.0116
MALMO 481|19|0 26441|667|4 1.814 [0.995,3.307] 0.052 0.012
SINAI 176|6|0 4723|213|6 0.815 [0.361,1.844] 0.62 0.0215
FinnGen 9357|249|1 119618|2693|15 1.182 [1.004,1.392] 0.044 0.0087
META 122452|3143|21 453886|10455|73 1.14 [1.089,1.193] 1.6e-08 0.0117
0.7
1
2
OR (95% CI)

j

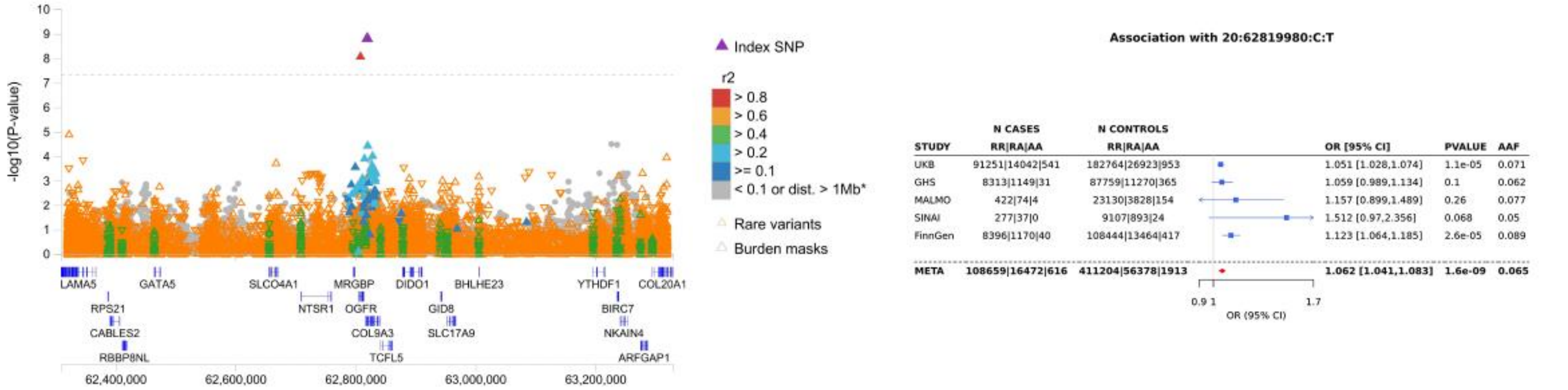
-log10(P-value)
Index SNP
r2
> 0.8
> 0.6
> 0.4
> 0.2
>= 0.1
< 0.1 or dist. > 1Mb*
Rare variants
Burden masks
LAMA5
GATA5
SLCO4A1
MRGBP
DIDO1
BHLHE23
YTHDF1
COL20A1
RPS21
NTSR1
OGFR
GID8
BIRC7
CABLES2
COL9A3
SLC17A9
NKAIN4
RBBP8NL
TCFL5
ARFGAP1
62,400,000
62,600,000
62,800,000
63,000,000
63,200,000
Association with 20:62819980:C:T
STUDY | N CASES RR|RA|AA | N CONTROLS RR|RA|AA | OR [95% CI] | PVALUE | AAF
UKB | 91251|14042|541 | 182764|26923|953 | 1.051 [1.028,1.074] | 1.1e-05 | 0.071
GHS | 8313|1149|31 | 87759|11270|365 | 1.059 [0.989,1.134] | 0.1 | 0.062
MALMO | 422|74|4 | 23130|3828|154 | 1.157 [0.899,1.489] | 0.26 | 0.077
SINAI | 277|37|0 | 9107|893|24 | 1.512 [0.97,2.356] | 0.068 | 0.05
FinnGen | 8396|1170|40 | 108444|13464|417 | 1.123 [1.064,1.185] | 2.6e-05 | 0.089
META | 108659|16472|616 | 411204|56378|1913 | 1.062 [1.041,1.083] | 1.6e-09 | 0.065
0.9 1
1.7
OR (95% CI)

k

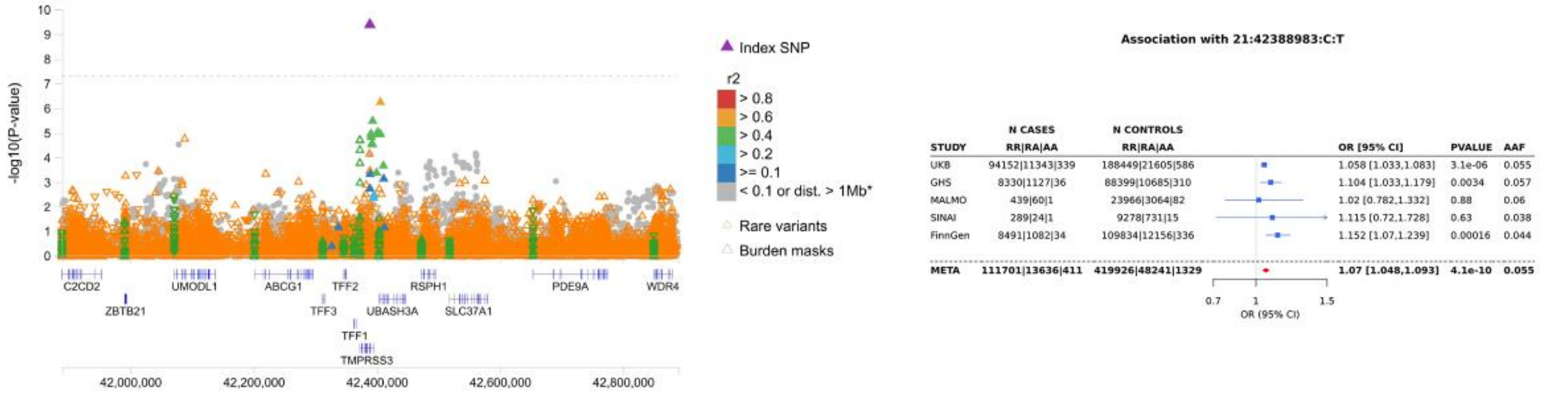
-log10(P-value)
Index SNP
r2
> 0.8
> 0.6
> 0.4
> 0.2
>= 0.1
< 0.1 or dist. > 1Mb*
Rare variants
Burden masks
C2CD2
UMODL1
ABCG1
TFF2
RSPH1
PDE9A
WDR4
ZBTB21
TFF3
UBASH3A
SLC37A1
TFF1
TMPRSS3
42,000,000
42,200,000
42,400,000
42,600,000
42,800,000
Association with 21:42388983:C:T
STUDY | N CASES RR|RA|AA | N CONTROLS RR|RA|AA | OR [95% CI] | PVALUE | AAF
UKB | 94152|11343|339 | 188449|21605|586 | 1.058 [1.033,1.083] | 3.1e-06 | 0.055
GHS | 8330|1127|36 | 88399|10685|310 | 1.104 [1.033,1.179] | 0.0034 | 0.057
MALMO | 439|60|1 | 23966|3064|82 | 1.02 [0.782,1.332] | 0.88 | 0.06
SINAI | 289|24|1 | 9278|731|15 | 1.115 [0.72,1.728] | 0.63 | 0.038
FinnGen | 8491|1082|34 | 109834|12156|336 | 1.152 [1.07,1.239] | 0.00016 | 0.044
META | 111701|13636|411 | 419926|48241|1329 | 1.07 [1.048,1.093] | 4.1e-10 | 0.055
0.7
1
1.5
OR (95% CI)

l

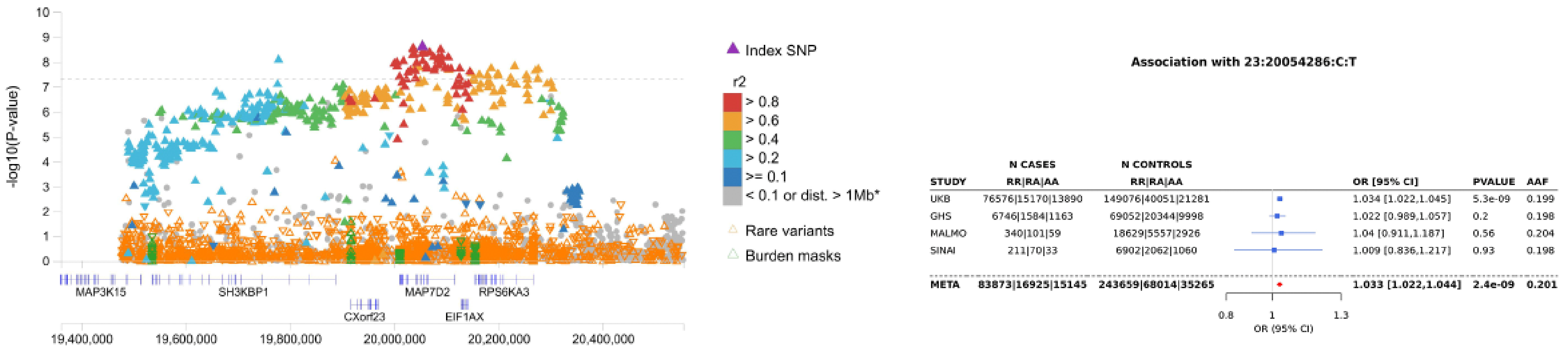
-log10(P-value)
Index SNP
r2
> 0.8
> 0.6
> 0.4
> 0.2
>= 0.1
< 0.1 or dist. > 1Mb*
Rare variants
Burden masks
MAP3K15
SH3KBP1
CXorf23
MAP7D2
EIF1AX
RPS6KA3
19,400,000
19,600,000
19,800,000
20,000,000
20,200,000
20,400,000
Association with 23:20054286:C:T
STUDY | N CASES RR|RA|AA | N CONTROLS RR|RA|AA | OR [95% CI] | PVALUE | AAF
UKB | 76576|15170|13890 | 149076|40051|21281 | 1.034 [1.022,1.045] | 5.3e-09 | 0.199
GHS | 6746|1584|1163 | 69052|20344|9998 | 1.022 [0.989,1.057] | 0.2 | 0.198
MALMO | 340|101|59 | 18629|5557|2926 | 1.04 [0.911,1.187] | 0.56 | 0.204
SINAI | 211|70|33 | 6902|2062|1060 | 1.009 [0.836,1.217] | 0.93 | 0.198
META | 83873|16925|15145 | 243659|68014|35265 | 1.033 [1.022,1.044] | 2.4e-09 | 0.201
0.8
1
1.3
OR (95% CI)

m

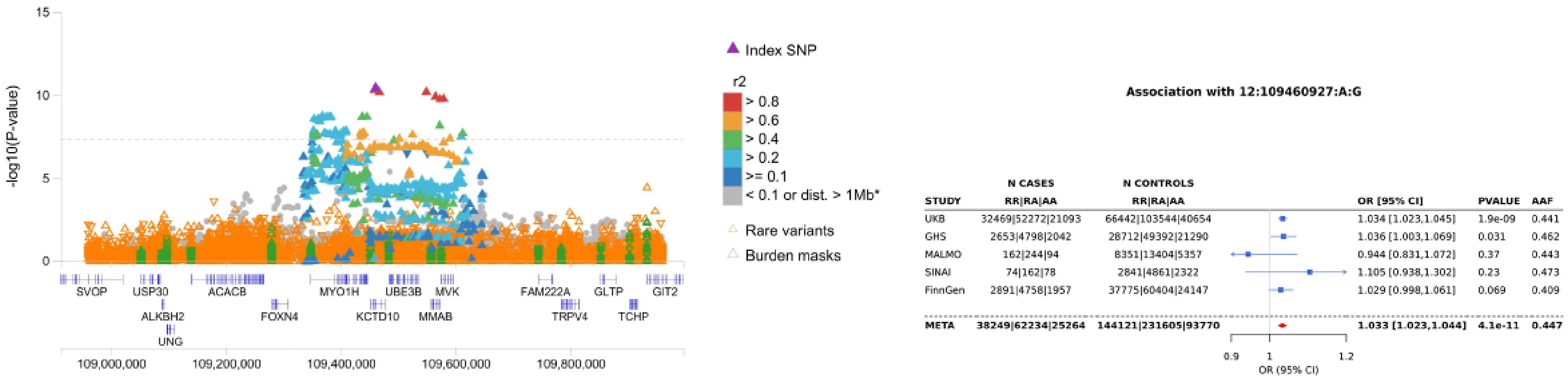
-log10(P-value)
Index SNP
r2
> 0.8
> 0.6
> 0.4
> 0.2
>= 0.1
< 0.1 or dist. > 1Mb*
Rare variants
Burden masks
SVOP
USP30
ALKBH2
UNG
ACACB
FOXN4
MYO1H
KCTD10
UBE3B
MMAB
MVK
FAM222A
TRPV4
GLTP
TCHP
GIT2
109,000,000
109,200,000
109,400,000
109,600,000
109,800,000
Association with 12:109460927:A:G
STUDY | N CASES RR|RA|AA | N CONTROLS RR|RA|AA | OR [95% CI] | PVALUE | AAF
UKB | 32469|52272|21093 | 66442|103544|40654 | 1.034 [1.023,1.045] | 1.9e-09 | 0.441
GHS | 2653|4798|2042 | 28712|49392|21290 | 1.036 [1.003,1.069] | 0.031 | 0.462
MALMO | 162|244|94 | 8351|13404|5357 | 0.944 [0.831,1.072] | 0.37 | 0.443
SINAI | 74|162|78 | 2841|4861|2322 | 1.105 [0.938,1.302] | 0.23 | 0.473
FinnGen | 2891|4758|1957 | 37775|60404|24147 | 1.029 [0.998,1.061] | 0.069 | 0.409
META | 38249|62234|25264 | 144121|231605|93770 | 1.033 [1.023,1.044] | 4.1e-11 | 0.447
0.9
1
1.2
OR (95% CI)

n

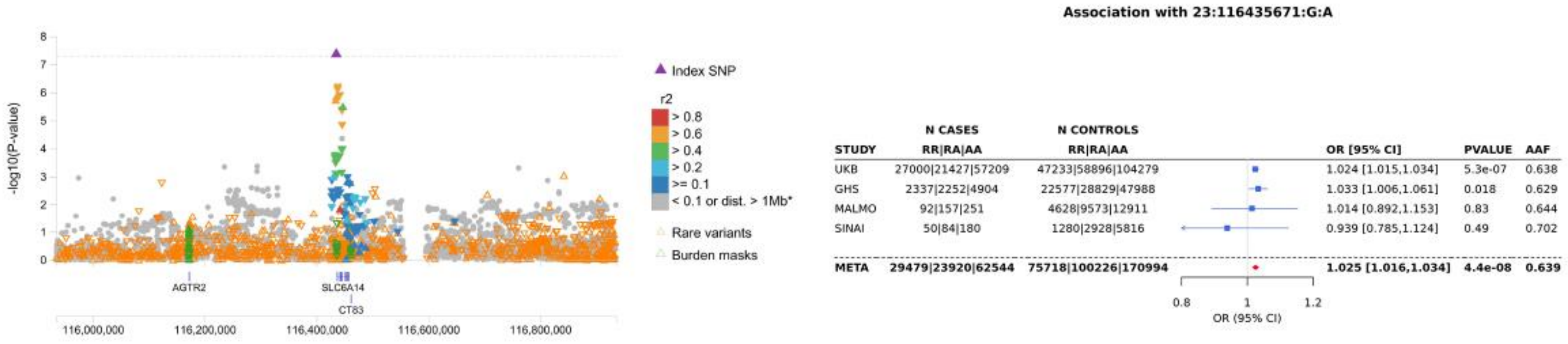
-log10(P-value)
Index SNP
r2
> 0.8
> 0.6
> 0.4
> 0.2
>= 0.1
< 0.1 or dist. > 1Mb*
Rare variants
Burden masks
AGTR2
SLC6A14
CT83
116,000,000
116,200,000
116,400,000
116,600,000
116,800,000
Association with 23:116435671:G:A
STUDY | N CASES RR|RA|AA | N CONTROLS RR|RA|AA | OR [95% CI] | PVALUE | AAF
UKB | 27000|21427|57209 | 47233|58896|104279 | 1.024 [1.015,1.034] | 5.3e-07 | 0.638
GHS | 2337|2252|4904 | 22577|28829|47988 | 1.033 [1.006,1.061] | 0.018 | 0.629
MALMO | 92|157|251 | 4628|9573|12911 | 1.014 [0.892,1.153] | 0.83 | 0.644
SINAI | 50|84|180 | 1280|2928|5816 | 0.939 [0.785,1.124] | 0.49 | 0.702
META | 29479|23920|62544 | 75718|100226|170994 | 1.025 [1.016,1.034] | 4.4e-08 | 0.639
0.8
1
1.2
OR (95% CI)

o

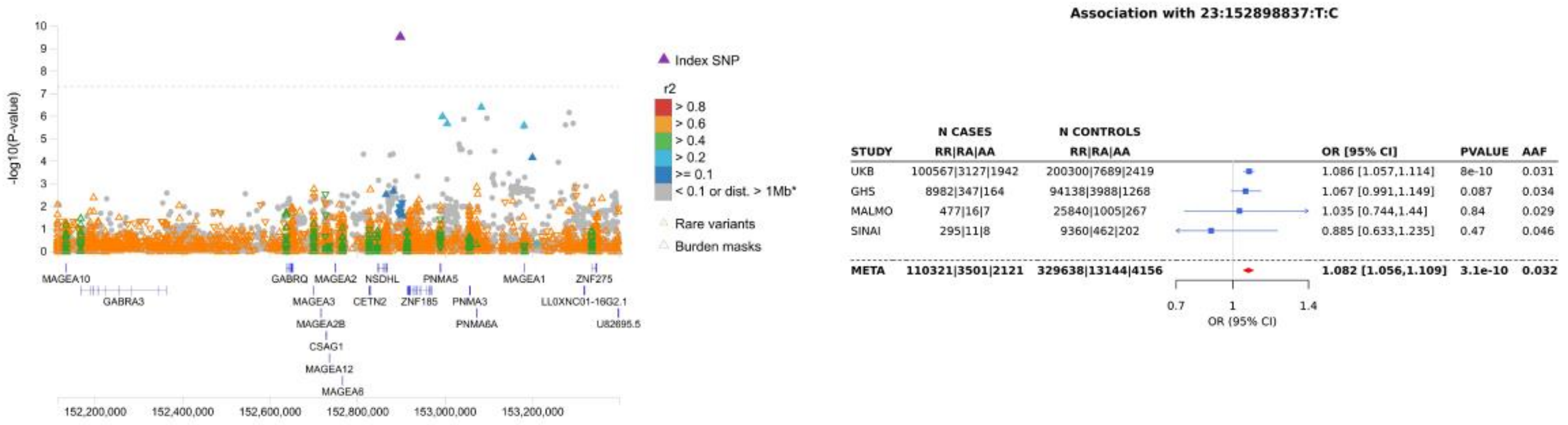
-log10(P-value)
Index SNP
r2
> 0.8
> 0.6
> 0.4
> 0.2
>= 0.1
< 0.1 or dist. > 1Mb*
Rare variants
Burden masks
MAGEA10
GABRA3
GABRQ
MAGEA2
MAGEA3
MAGEA2B
CSAG1
MAGEA12
MAGEA6
NSDHL
CETN2
ZNF185
PNMA5
PNMA3
PNMA6A
MAGEA1
ZNF275
LL0XNC01-16G2.1
U82695.5
152,200,000
152,400,000
152,600,000
152,800,000
153,000,000
153,200,000
Association with 23:152898837:T:C
STUDY | N CASES RR|RA|AA | N CONTROLS RR|RA|AA | OR [95% CI] | PVALUE | AAF
UKB | 100567|3127|1942 | 200300|7689|2419 | 1.086 [1.057,1.114] | 8e-10 | 0.031
GHS | 8982|347|164 | 94138|3988|1268 | 1.067 [0.991,1.149] | 0.087 | 0.034
MALMO | 477|16|7 | 25840|1005|267 | 1.035 [0.744,1.44] | 0.84 | 0.029
SINAI | 295|11|8 | 9360|462|202 | 0.885 [0.633,1.235] | 0.47 | 0.046
META | 110321|3501|2121 | 329638|13144|4156 | 1.082 [1.056,1.109] | 3.1e-10 | 0.032
0.7
1
1.4
OR (95% CI)

Supplementary Figure 2: Regional plots for novel common (MAF ≥ 1%) loci identified in hearing loss meta-analysis and forest plots corresponding to the index variant at each novel locus. (Panels a through o, pages 4-11.)

Supplementary Figure 3.

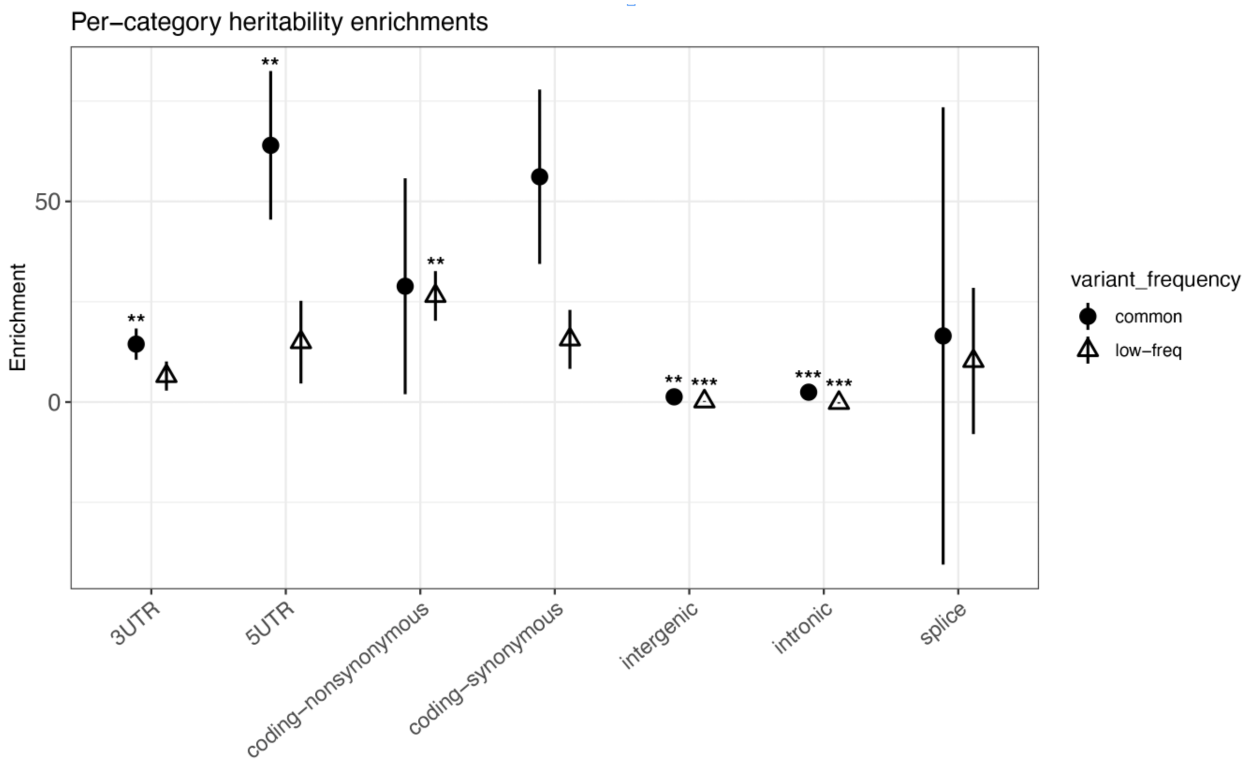

Supplementary Figure 3: Heritability enrichments from stratified LD score regression analysis. Total SNP heritability for seven functional categories, each further stratified by MAF into a common variant (CV, MAF $\geq$ 0.05) and low-frequency variant (LFV, 0.001 $\leq$ MAF<0.05) bin, was estimated and enrichments for these categories was calculated (proportion heritability / proportion variants). Plotted are the resulting enrichments (common variant bins shown as solid circles and low-frequency variant bins as open triangles) with standard errors. Significant enrichments and depletions are denoted by asterisks (Bonferroni-corrected p<0.05 = *; p<0.01 = **; p<0.001 = ***)

Supplementary Figure 4.

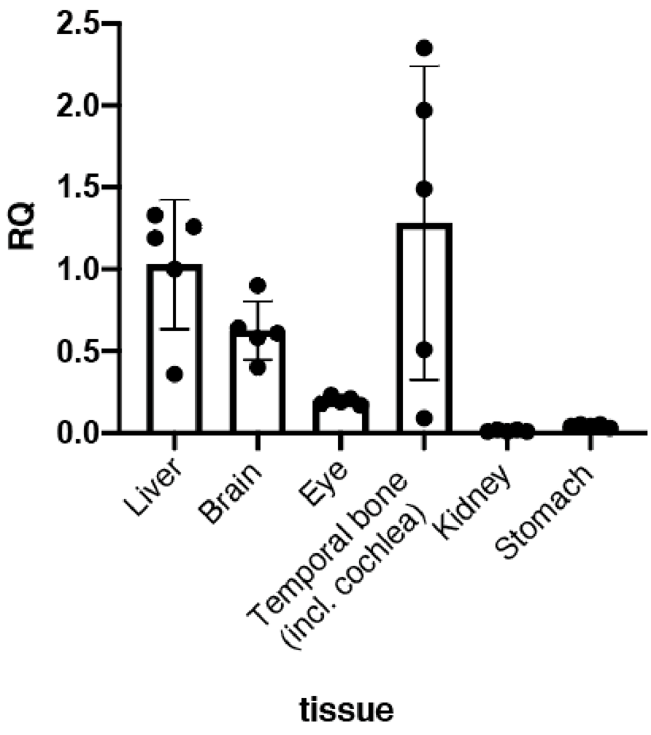

Supplementary Figure 4: KLHDC7b is expressed in tissue within the temporal bone. RNA was extracted from different organs, and qPCR was performed using a primer/probe combination for KLHDC7b, and one for Drosha as a housekeeping control. Data for each organ was normalized to the housekeeping control and then normalized to liver expression. Expression in cochlea and brain were not significantly different from expression in liver, which was relatively high. RQ = relative quantification compared to liver (see methods).

Supplementary Methods

Phenotype definition:

Hearing loss in GHS, MALMO and SINAI was defined using ICD-10 codes: cases were individuals who had (1) a problem-list entry of the ICD-10 diagnosis code (H903-H908, H911, H919), (2) an inpatient hospitalization-discharge ICD-10 diagnosis code, or (3) an encounter ICD-10 diagnosis code entered for 2 separate outpatient visits on separate calendar days. Controls were individuals without any of the criteria for case definition. Individuals were excluded if they had the relevant ICD-10 code associated with only one outpatient encounter. We also excluded from controls any individuals who were cases for ICD-10 Q16 (congenital malformations of ear causing hearing impairment) or ICD-10 H931 (tinnitus).

In UKB, hearing loss was defined using ICD-10 codes and self-reports based on two questions: "Do you have any difficulty with your hearing?" (Field: 2247) and "Do you find it difficult to follow a conversation if there is background noise (such as TV, radio, children playing)?" (Field: 2257). Self-reported cases were individuals who (1) answered 'yes' to both questions or (2) were completely deaf or (3) were a case for any of the following ICD-10 codes: H903-H908, H911, H919. Phenotype definition of ICD10-based cases required one or more of the following: a) ≥ 1 diagnosis in inpatient Health Episode Statistics (HES) records, b) a cause-of-death diagnosis in death registry, c) ≥ 2 diagnoses in outpatient data (READ codes mapped to ICD10). ICD-based controls were individuals who did not meet the case critera, and were not cases for ICD-10 Q16 and ICD-10 H931. To obtain the overall cases in the analysis, self-reported and ICD-based cases were combined. Controls for the overall analysis were defined as individuals who (1) answered 'No' to both self-report hearing loss questions and, (2) did not report that they were deaf and (3) did not meet the criteria for ICD-based case definition and (4) did not have tinnitus

based on ICD-10 (H931) or self-reported tinnitus (Field ID: 4803, 4814 and self-reported from verbal interview).

Genotyping

For SINAI and MALMO, DNA from participants was genotyped on the Global Screening Array (GSA), and for GHS genotyping was done on either the Illumina OmniExpress Exome (OMNI) or GSA. These cohorts were imputed to the TOPMed (GHS) or the HRC (MALMO, SINAI) reference panels (stratified by array for GHS) using the University of Michigan Imputation Server or the TOPMed Imputation Server (URLs). Prior to imputation, we retained variants that had a MAF ≥ 0.1%, missingness < 1% and HWE $P > 10^{-15}$. Following imputation for GHS, data from the OMNI and GSA datasets were merged for subsequent association analyses, which included an OMNI/GSA batch covariate in addition to other covariates described below. UKB DNA samples were genotyped as described previously[1] using the Applied Biosystems UK BiLEVE Axiom Array (N=49,950) or the closely related Applied Biosystems UK Biobank Axiom Array (N=438,427). Genotype data for variants not included in the arrays were imputed using three reference panels (Haplotype Reference Consortium, UK10K and 1000 Genomes Project phase 3) as described previously[1]. FinnGen data were derived from a custom Axiom array and imputed into the FinnGen SISu v3 reference panel (https://finngen.gitbook.io/documentation/methods/genotype-imputation/sisu-reference-panel) .

Exome sequencing

High coverage whole exome sequencing was performed at the Regeneron Genetics Center as previously described[2,3]. NimbleGen probes (VCRome) or a modified version of the xGen design from Integrated DNA Technologies (IDT) were used for target sequence capture,

and sequencing was performed using 75 bp paired-end reads on Illumina v4 HiSeq 2500 or NovaSeq instruments to a coverage depth greater than 20x at at least 85% of targeted bases in 96% of VCRome samples, and at least 90% of targeted bases in 99% of IDT samples. Sequence read alignment and variant calling was based on the GRCh38 Human Genome reference sequence. Ensembl v85 gene definitions were used to determine variants' functional impacts. Predicted LOF genetic variants included (a) insertions or deletions resulting in a frameshift, (b) insertions, deletions or single nucleotide variants resulting in the introduction of a premature stop codon or in the loss of the transcription start site or stop site, and (c) variants in donor or acceptor splice sites. Missense variants were classified for likely functional impact according to the number of *in silico* prediction algorithms that predicted deleteriousness using SIFT, Polyphen2_HDIV and Polyphen2_HVAR, LRT and MutationTaster. We aggregated rare variants for gene burden testing as previously described[4]. Briefly, rare variants were collapsed by gene region, such that individuals who are homozygous reference for all variants are considered homozygous reference, heterozygous carriers of any aggregated variant are considered heterozygous, and only minor allele homozygotes for an aggregated variant are considered as minor allele homozygotes. Genotypes were not phased to consider compound heterozygotes in burden testing. For each gene, we considered four categories of aggregates: a strict burden of rare pLOFs and three more permissive burden of rare pLOFs and missense variants. The missense variants in the burden aggregates were defined as 'strict deleterious missense' if predicted deleterious by 5/5 prediction algorithms (SIFT, Polyphen2_HDIV, Polyphen2_HVAR, LRT, MutationTaster), and 'deleterious missense' if predicted deleterious by at least 1/5[4]. For each of these groups, we considered five separate burden masks per gene, based on the frequency of the alternative allele of the variants that were screened in that group: MAF ≤ 1%, MAF ≤ 0.1%, MAF

≤ 0.01%, MAF ≤ 0.001%, and singletons only. For the purposes of gene burden testing, the singleton mask includes minor allele homozygotes if no other variant carriers are observed in the dataset. We conducted further QC of associated variants *post-hoc*, based on mappability statistics from read alignment.

Genetic association analyses

Association analyses in each study were performed using the Firth logistic mixed model regression test implemented in REGENIE[5]. We included in step 1 of REGENIE (i.e. prediction of individual trait values based on the genetic data) directly genotyped variants with a minor allele frequency (MAF) > 1%, < 10% missingness, Hardy-Weinberg equilibrium test $P > 10^{-15}$ and linkage-disequilibrium (LD) pruning (1000 variant windows, 100 variant sliding windows and $r^2 < 0.9$). The association model used in step 2 of REGENIE included as covariates (i) age, age^2, sex, age-by-sex and age^2-by-sex; (ii) 10 ancestry-informative principal components (PCs) derived from the analysis of a set of LD-pruned (50 variant windows, 5 variant sliding windows and $r^2 < 0.5$) common variants from the array (imputed for the GHS study) data generated separately for each ancestry; (iii) an indicator for exome sequencing batch (GHS: three batches; UKB: six batches); and (iv) 20 PCs derived from the analysis of exome variants with minor allele count ≤ 20 and MAF < 1% also generated separately for each ancestry.

We determined continental ancestries by projecting each sample onto reference principal components calculated from the HapMap3 reference panel. Briefly, we merged our samples with HapMap3 samples and kept only SNPs in common between the two datasets. We further excluded SNPs with MAF < 10%, genotype missingness > 5% or Hardy-Weinberg Equilibrium test $P < 10^{-5}$. We calculated PCs for the HapMap3 samples and projected each of our samples onto those PCs. To assign a continental ancestry group to each non-HapMap3 sample, we trained a kernel density

estimator (KDE) using the HapMap3 PCs and used the KDEs to calculate the likelihood of a given sample belonging to each of the five continental ancestry groups. When the likelihood for a given ancestry group was > 0.3, the sample was assigned to that ancestry group. When two ancestry groups had a likelihood > 0.3, we arbitrarily assigned AFR over EUR, Admixed American (AMR) over EUR, AMR over East Asian (EAS), South Asian (SAS) over EUR, and AMR over AFR. Samples were excluded from analysis if no ancestry likelihoods were > 0.3, or if more than three ancestry likelihoods were > 0.3. Results were subsequently meta-analyzed across studies and ancestries using an inverse variance-weighted fixed-effects meta-analysis using an inverse variance-weighted model in METAL[6].

Finemapping and follow-on genetic analyses

LD score (LDSC) regression[7] was used to assess inflation (LDSC intercept) in our accounting for polygenic signal. We used LD scores calculated using genotyped or imputed variants INFO>0.3 and MAF>0.5% from 10,000 randomly chosen subjects from UKB, and restricted our analysis to HapMap3 variants.

We defined previously associated loci by their index variants reported in previous hearing loss GWAS, and excluded 1 Mb regions surrounding them in the identification of novel loci in our analysis. We defined genome-wide significant loci in our analysis by linkage disequilibrium ($r^2 > 0.1$) with lead variants.

Forward stepwise conditional analyses were carried out in every locus with GCTA-COJO using summary statistics and a UK Biobank subsample LD reference panel. Independent associations were determined using a joint P-value threshold of 1×10^{-5} and r^2 cutoff of 0.9.

Bayesian causal variant inference was conducted in available individual level data using FINEMAP[8].

Rare variant association analyses conditional on the common variant signal were carried out for four loci with both common (MAF ≥ 0.01) and single rare variant (MAF < 0.01) genome-wide significant signals. For these loci, the dosages for variants representing the common variant signal were included as covariates in REGENIE logistic regression. The specific variants that best captured the common variant signal were ascertained through fine mapping analyses (FINEMAP 80% credible sets when available, or GCTA-COJO-identified independent [$r^2 < 0.9$] significant [joint P-value$<1 \times 10^{-4}$] variants). Burden analyses conditional on rare variants were carried out for five genes with significant single rare variants in addition to their burden associations. For these genes, conditional burden association statistics were obtained through inclusion of dosages for the top (most significant) single rare variant in each gene. Conditional analyses were performed for each cohort using REGENIE's Firth-corrected logistic regression and the resulting summary statistics were meta-analyzed as described above.

Assessment of heterozygous effects used association analyses excluding homozygotes as well as individuals potentially carrying compound heterozygous mutations (CHMs) called as follows. Available unphased genotype array data and genetically inferred pedigree structures determined by PRIMUS[9] were used to create a phased genetic scaffold with the program MakeScaffold (https://github.com/odelaneau/makeScaffold). The scaffold and the unphased exome data where then provided to SHAPEIT4 (https://github.com/odelaneau/shapeit4)[9,10] to generate phased exome variant calls. We then identified pairs of exome sequenced variants (MAF $< 2\%$ and MAC > 1) within the same person and gene as potential CHMs (pCHM), and determined

them to be in cis, trans or unknown based on the phased exome data. Trans or unknown-phase pCHMs were excluded.

Power curves were generated by specifying risk allele frequency (RAF), ranging from $1x10^{-6}$ to 1, and the numbers of cases and controls in our meta-analysis, and then determining which genotype relative risk (GRR) values provide 80 and 50 percent power given the risk allele frequency. *z*-score non-centrality parameters (NCP, i.e. expected values for Wald association test statistics) for a case-control study was obtained following Zaitlen et. al.[11], power was obtained using the `pnorm(u=NCP)` and `qnorm()` R functions, and GRRs for 50 and 80% power curves given RAFs were estimated numerically.

Heritability derived from variants in specific functional categories and minor allele frequency bins (in an approach similar to stratified LD score regression) was estimated using partitioned LD score regression (LDSC) of hearing loss association statistics on LD scores. In order to capture LD from both low-frequency and common variants, a reference panel (N = 10,000 samples) generated from the merging of UK Biobank (European-ancestry) imputed and exome data was used. Reference panel variants were annotated using an internal pipeline and LD scores with respect to seven functional categories (coding-synonymous, coding-nonsynonymous, 5-prime-UTR, 3-prime-UTR, splice site, intronic, and intergenic), each split into a common (MAF > 0.05) and low-frequency (0.001 < MAF ≤ 0.05) variant bin, were calculated. Variants used to calculate LD scores were filtered for false positives identified through support vector machine learning of QC metrics, and both reference panel variants and summary statistics were restricted to those with MAF > 0.001. Since our categories have a very small degree of overlap, with approximately 0.01 percent of variants falling into more than one category, reported per-category enrichment results were taken from the .results file (supplementary table 11) provided by LDSC

when using the –overlap-annot flag. As LDSC does not provide per-category h2 estimates when using this flag, however, we used per-category heritability estimates taken from the .log file from non-overlap-annot runs to calculate h^2_{CV} and h^2_{LFV} each as the sum of the relevant common and low-frequency variant category heritabilities.

For genes overlapping genome-wide significant hearing loss loci, coloc2[12] was used to assess evidence for co-localization between our hearing loss GWAS and GTEx (release v8) eQTL derived from 48 tissues (URLs). GWAS and eQTL summary statistics for all common (MAF > 0.01) variants within each gene's cis-region were used as input to coloc2, which then estimates posterior probabilities for five hypotheses (H_0, no association; H_1, GWAS association only; H_2, eQTL association only; H_3, both but not co-localized; H_4, both and co-localized) given the association statistics and prior probabilities estimated from the observed data. Genes with posterior probability of $H_4 \geq 0.5$ were determined as having evidence for co-localization.

Single-Cell RNA Sequencing and Analysis

Cochlea and utricles from C57BL/6 mice at post-natal day 7 were micro-dissected and dissociated via incubation in 0.05% trypsin at 37°C for 20 - 30 min. Four volumes of 5% FBS in DMEM/F12 with a final concentration of DNAse (LK003170) greater than or equal to 100 Kunitz per mL was added to inactivate any remaining trypsin. The tissues were then triturated 20 times and passed through a 40-µm strainer to eliminate residual aggregates / clumps of cells. Cells were counted then resuspended in 0.04% BSA in PBS at a concentration of 200 cells / uL.

Single cells suspended in PBS with 0.04% BSA were loaded on a Chromium Single Cell Instrument (10xGenomics). RNAseq libraries were prepared using the Chromium Single Cell 3′Library, Gel Beads & Multiplex Kit (10x Genomics). Paired-end sequencing was performed on

an Illumina NextSeq 500 (Read 1 26-bp for unique molecular identifier, UMI, and cell barcode, 8-bp i7 sample index, 0-bp i5, and Read 2 55-bp transcript read). Cell Ranger Single-Cell Software Suite (10X Genomics, v2.0.0) was used to perform sample de-multiplexing, alignment, filtering, and UMI counting. The Mouse MM10 Genome assembly and UCSC gene models were used for alignment. CellRanger output was processed using Seurat V3.2 (URLs)[13]. Cells with number of genes detected of less than 500 or over 15000, or UMI ratio of mitochondria encoded genes vs. all genes over 0.15 were removed. Data normalization and scaling for each cell were achieved by using Seurat global-scaling "LogNormalize" method. To avoid potential sample-to-sample variation caused by technical variation at various experiment steps, we employed Seurat's data integration pipeline to merge all cochlea and utricle samples into a single Seurat object for downstream analysis. Statistically significant principal components identified by Seurat's "RunPCA" function were used to define the dimensions for the UMAP nonlinear dimensionality reduction analysis, which then visualized the cells on a 2D UMAP plot. Unsupervised clustering, via Seurat's "FindClusters" method (resolution = 0.6), identified groups of molecularly distinct cells on the plot. Clusters in the UMAP plot were annotated based on cluster-specific genes identified via Seurat's "FindAllMarkers" (min.pct = 0.25, thresh.use = 0.25) function. The expression of cluster marker genes as well as canonical cell type-specific genes were used to label the cell type for each cluster.

Mouse cochlea dissection for KLHDC7B quantitation:

There were five biological replicates and three technical replicates of each sample. Cochlea were dissected from B6.CAST-Cdh23Ahl$^{+/WT}$ mice ears. Both cochleae were pooled per mouse from five female mice aged 11-28 weeks. Animals were sacrificed by carbon dioxide inhalation

followed by immediate removal of the organs in question, which were stored in RNA later. Cochleae were pierced at the apex and oval window, then gently flushed with ~100 µl of RNA later, frozen on dry ice and stored at -80 degrees Celsius, while other organs were placed in RNA later and frozen at -20 degrees Celsius until RNA extraction. The vestibular system was included with the cochlea.

RNA extraction and analysis:

Tissue/Cells were homogenized in TRIzol, and chloroform was used for phase separation. The aqueous phase, containing total RNA, was purified using MagMAX™-96 for Microarrays Total RNA Isolation Kit (Ambion by Life Technologies) according to manufacturer's specifications. Genomic DNA was removed using RNase-Free DNase Set (Qiagen).

mRNA was reverse-transcribed into cDNA using SuperScript® VILO™ Master Mix (Invitrogen by Life Technologies). cDNA was amplified with the SensiFAST Probe Lo-ROX (Meridian) using the 12K Flex System (Applied Biosystems). An endogenous control gene was used to normalize any cDNA input differences. The KLHDC7b primer/probe combination was forward: GGTGGCCCTGGATGGAATG, reverse: TCTGTGCGTGGGTCATAGC, probe: TTTATGCCATTGGTGGCGAGTGC. The Drosha housekeeping control spanned exons 34-35 and was acquired from Thermofisher (Mm01310009_m1, catalogue number 4331182). Data is reported as the comparative CT method using ΔΔCT. The ΔCt = Klhdc7b – Drosha (housekeeping transcript), ΔΔCt = ΔCt – ΔCt reference sample; Relative quantification (RQ) = 2^ - ΔΔCt.

Supplementary References

1. Bycroft, C. *et al.* The UK Biobank resource with deep phenotyping and genomic data. *Nature* **562**, 203–209 (2018).
2. Dewey, F. E. *et al.* Distribution and clinical impact of functional variants in 50,726 whole-exome sequences from the DiscovEHR study. *Science* **354**, aaf6814 (2016).
3. Van Hout, C. V. *et al.* Exome sequencing and characterization of 49,960 individuals in the UK Biobank. *Nature* **586**, 749–756 (2020).
4. Kosmicki, J. A. *et al.* Pan-ancestry exome-wide association analyses of COVID-19 outcomes in 586,157 individuals. *Am. J. Hum. Genet.* **108**, 1350–1355 (2021).
5. Mbatchou, J. *et al.* Computationally efficient whole-genome regression for quantitative and binary traits. *Nat. Genet.* (2021) doi:10.1038/s41588-021-00870-7.
6. Willer, C. J., Li, Y. & Abecasis, G. R. METAL: fast and efficient meta-analysis of genomewide association scans. *Bioinformatics* **26**, 2190–2191 (2010).
7. Bulik-Sullivan, B. K. *et al.* LD Score regression distinguishes confounding from polygenicity in genome-wide association studies. *Nat. Genet.* **47**, 291–295 (2015).
8. Benner, C. *et al.* FINEMAP: efficient variable selection using summary data from genome-wide association studies. *Bioinformatics* **32**, 1493–1501 (2016).
9. Staples, J. *et al.* PRIMUS: rapid reconstruction of pedigrees from genome-wide estimates of identity by descent. *Am. J. Hum. Genet.* **95**, 553–564 (2014).
10. Delaneau, O., Zagury, J.-F., Robinson, M. R., Marchini, J. L. & Dermitzakis, E. T. Accurate, scalable and integrative haplotype estimation. *Nat. Commun.* **10**, 5436 (2019).

11. Zaitlen, N., Paşaniuc, B., Gur, T., Ziv, E. & Halperin, E. Leveraging genetic variability across populations for the identification of causal variants. *Am. J. Hum. Genet.* **86**, 23–33 (2010).

12. Dobbyn, A. *et al.* Landscape of Conditional eQTL in Dorsolateral Prefrontal Cortex and Co-localization with Schizophrenia GWAS. *Am. J. Hum. Genet.* **102**, 1169–1184 (2018).

13. Butler, A., Hoffman, P., Smibert, P., Papalexi, E. & Satija, R. Integrating single-cell transcriptomic data across different conditions, technologies, and species. *Nat. Biotechnol.* **36**, 411–420 (2018).
