# Supplementary material for: Population-scale analysis of common and rare genetic variation associated with hearing loss in adults
Source: Commun Biol. 2022 Jun 3;5:540. doi: 10.1038/s42003-022-03408-7 (PMC9166757; doi:10.1038/s42003-022-03408-7)
Supplement: Supplementary file 3 — Description of Additional Supplementary Files [file 42003_2022_3408_MOESM3_ESM.pdf]

Description of Additional Supplementary Files

File name: Supplementary Data 1-11
Description:

Supplementary Data 1: Counts of cases and controls included in hearing loss meta-analysis from each cohort.

Supplementary Data 2: **Index variants at all loci (P<5E-08) associated with hearing loss in the meta-analysis.** The direction of the effect in each single study (in the order: MALMO, UKB, GHS, SINAI, FinnGen) in the meta-analysis is given in the 'Direction' column where '+' indicates increased risk, '-' indicates decreased risk and '?' indicates that the variant was not present or tested in this dataset. The 'coloc2' Genes column lists genes at each locus, if any, that were highlighted by colocalisation analyses (see supplementary table 4 for more details).

Supplementary Data 3: Fine-mapping analyses in autosomal genome-wide significant loci. Shown are FINEMAP results for loci that were genome-wide significant in RGC data, and GCTA-COJO results for loci that were genome-wide significant after meta-analysis with FinnGen data.

Supplementary Data 4: **GWAS-eQTL co-localization results.** Genes at genome-wide significant loci that show co-localization (posterior probability for co-localization, PPH4>=0.5) in at least one GTEx eQTL tissue. Genes at novel hearing loss loci are highlighted in green. An asterisk marks genes with direct or indirect evidence for hearing loss in model organisms (LMO7: Du et al, 2019 PubMedID 30850599; SPTBN1: Liu et al, 2019 PMID 31001589; CLDN7: Kudo et al, 2018 PMID 29368643, Li et al, 2018 PMID 28834538). Columns R to V represent results from the intersection of coloc-identified genes with single-cell gene expression data from the mouse inner ear: The 'Average % positive' column is the average of the percetage of positive cells (defined as having a non-zero unique molecular identifier (UMI) count) across the 26 cell types in the experiment; 'Max. % positive' column is the maximum percentage of positive cells across the 14 cell types; '# Cell types' and 'Positive cell types' columns give the numbers and names of cell types (out of 14) that had at least 20% positive cells for each gene, reespectively; A 'yes' in the 'Ear expression summary' is given if either 'Average % positive' and 'Max % positive' are ≥ 0.2, and a 'no' is given if both columns are < 0.2. * in column R, Cyp26c1 was not detected in any cells in RNAseq and was removed from analysis during UMI transformation/normalization.

Supplementary Data 5: **Expression of genes highlighted by coloc2 in inner ear cell-types from mouse single-cell RNAseq data.** Column labels ending with 'pct.positive' indicate the percetage of cells in a given cell type that have a non-zero unique molecular identifier (UMI) count. Column labels ending with 'mean' provide the mean of the normalized UMI per cell type. AVG.pct.positive and MAX.pct.positive are the percent of cells positive across the entire experiment (all cell types) and the maximum across cell types, respectively. GER = greater epithelial ridge.

Supplementary Data 6: Rare (MAF<1%) variants (P<5E-08) associated with hearing loss in meta-analysis. The yellow colored rows indicate coding variants. Variants that were present only in FinnGen and not in any other cohorts are not shown. The direction of the effect in each single study (in the order: MALMO, UKB, GHS, SINAI, FinnGen) in the meta-analysis is given in the 'Direction' column where '+' indicates increased risk, '-' indicates decreased risk and '?' indicates that the variant was not present or tested in this dataset. NA: Not applicable (no significant common variant signal was identified in the locus); NT: not tested in conditional analyses.

Supplementary Data 7: **Gene burden associations (P<5E-08) from the meta-analysis of hearing loss.** The 'Variant grouping' column provides a description of how variants were aggregated within each gene. Variants are called 'strict deleterious' and 'deleterious' if they were predicted deleterious by 5 different algorithms and at least 1 of 5 algorithms, respectively (see Methods for details). The direction of the effect in each single study (in the order: MALMO, UKB, GHS, SINAI, FinnGen) in the meta-analysis is given in the 'Direction' column where '+' indicates increased risk, '-' indicates decreased risk and '?' indicates that the variant was not present or tested in this dataset. The "Conditional P-value" refers to the p-value after conditioning on the rare variant signals in that gene (P-value<5E-08; refer to Table S6).

Supplementary Data 8: Rare variant and burden results after conditioning on common variant associations prioritized by FINEMAP (80% credible set) or identified by GCTA-COJO variants (joint P-value<1 × 10-4).

Supplementary Data 9: Variants included in the significant burden tests (Table S6) and their frequencies in hearing loss cases and controls.

Supplementary Data 10: **Association of variants/burden in GJB2 and SLC26A5 excluding homozygous and compound heterozygous carriers across GHS, UKB, MALMO, SINAI and the meta-analysis of all four cohorts.** The odds ratios (OR) and P-values for analysis that includes compound heterozygous and homozygous individuals (using only exome-sequenced genotypes) are also provided for comparison.

Supplementary Data 11: Results from LD score regression heritability analysis partitioned by functional categories and minor allele frequency (MAF). CV = common variants; LFV = low frequency variants; Enrichment = Proportion of h2 / Proportion of SNPs.
