# Supplementary material for: Population-scale analysis of common and rare genetic variation associated with hearing loss in adults
Source: Commun Biol. 2022 Jun 3;5:540. doi: 10.1038/s42003-022-03408-7 (PMC9166757; doi:10.1038/s42003-022-03408-7)
Supplement: Supplementary file 5 — Reporting Summary [file 42003_2022_3408_MOESM5_ESM.pdf]

nature portfolio

Corresponding author(s): Giovanni Coppola

Last updated by author(s): 02/28/2022

Reporting Summary

Nature Portfolio wishes to improve the reproducibility of the work that we publish. This form provides structure for consistency and transparency in reporting. For further information on Nature Portfolio policies, see our Editorial Policies and the Editorial Policy Checklist.

Statistics

For all statistical analyses, confirm that the following items are present in the figure legend, table legend, main text, or Methods section.

n/a	Confirmed	
☐	☒	The exact sample size (n) for each experimental group/condition, given as a discrete number and unit of measurement
☒	☐	A statement on whether measurements were taken from distinct samples or whether the same sample was measured repeatedly
☐	☒	The statistical test(s) used AND whether they are one- or two-sided<br>*Only common tests should be described solely by name; describe more complex techniques in the Methods section.*
☐	☒	A description of all covariates tested
☐	☒	A description of any assumptions or corrections, such as tests of normality and adjustment for multiple comparisons
☐	☒	A full description of the statistical parameters including central tendency (e.g. means) or other basic estimates (e.g. regression coefficient) AND variation (e.g. standard deviation) or associated estimates of uncertainty (e.g. confidence intervals)
☐	☒	For null hypothesis testing, the test statistic (e.g. F, t, r) with confidence intervals, effect sizes, degrees of freedom and P value noted<br>*Give P values as exact values whenever suitable.*
☐	☒	For Bayesian analysis, information on the choice of priors and Markov chain Monte Carlo settings
☒	☐	For hierarchical and complex designs, identification of the appropriate level for tests and full reporting of outcomes
☐	☒	Estimates of effect sizes (e.g. Cohen's d, Pearson's r), indicating how they were calculated

Our web collection on statistics for biologists contains articles on many of the points above.

Software and code

Policy information about availability of computer code

Data collection	NA
Data analysis	Software used for data analysis included Regenie (version 0.0.23), FINEMAP, coloc2, GCTA , MakeScaffold, SHAPEIT4, R.

For manuscripts utilizing custom algorithms or software that are central to the research but not yet described in published literature, software must be made available to editors and reviewers. We strongly encourage code deposition in a community repository (e.g. GitHub). See the Nature Portfolio guidelines for submitting code & software for further information.

Data

Policy information about availability of data

All manuscripts must include a data availability statement. This statement should provide the following information, where applicable:

- Accession codes, unique identifiers, or web links for publicly available datasets
- A description of any restrictions on data availability
- For clinical datasets or third party data, please ensure that the statement adheres to our policy

All whole-exome sequencing, genotyping chip, and imputed sequence for UKB described in this report are publicly available to registered researchers via the UK Biobank data access protocol. Additional information about registration for access to the data is available at http://www.ukbiobank.ac.uk/register-apply/. Further information about the whole exome sequence is available at http://www.ukbiobank.ac.uk/wp-content/uploads/2019/03/Access_064-UK-Biobank-50k-Exome-Release-FAQ-v3.pdf Detailed information about the chip and imputed sequence is available at: http://www.ukbiobank.ac.uk/wp-content/uploads/2018/03/UKB-Genotyping-and-Imputation-Data-Release-FAQ-v3-2-1.pdf. Geisinger DiscovEHR, Malmo Diet and Cancer study and Mt. Sinai Biome exome sequencing and genotyping data can be made available to qualified, academic, non-commercial researchers upon request via a Data Transfer Agreement with the respective institutions. Summary statistics for FinnGen r3 can be downloaded from https://www.finngen.fi/en/access_results.

Field-specific reporting

Please select the one below that is the best fit for your research. If you are not sure, read the appropriate sections before making your selection.

☒ Life sciences ☐ Behavioural & social sciences ☐ Ecological, evolutionary & environmental sciences

For a reference copy of the document with all sections, see nature.com/documents/nr-reporting-summary-flat.pdf

Life sciences study design

All studies must disclose on these points even when the disclosure is negative.

Sample size	No statistical methods were used to determine sample size. For human genetic analyses, we aimed to maximize the power for detecting associations with rare variants by maximizing the number of hearing loss cases. In order to have a balance between optimal power and phenotype quality, we combined two self-reported hearing loss phenotypes along with ICD code defined hearing loss.
Data exclusions	For the case control analysis, individuals in controls who had an icd-code or self-reported tinnitus or congenital defects of the ear were excluded. In cases, individuals who reported as having only hearing difficulties or only hearing difficulties with background noise were excluded from the analysis (cases had to have reported having both issues).
Replication	We included 5 cohorts in total and reported results that were genome-wide significant in a meta-analysis of all 5 cohorts.
Randomization	For genetic analysis, cases and controls assignments were based on self-reported or on electronic health record information.
Blinding	This is not relevant as this study is not experimental.

Reporting for specific materials, systems and methods

We require information from authors about some types of materials, experimental systems and methods used in many studies. Here, indicate whether each material, system or method listed is relevant to your study. If you are not sure if a list item applies to your research, read the appropriate section before selecting a response.

Materials & experimental systems

n/a	Involved in the study	
☒	☐	Antibodies
☒	☐	Eukaryotic cell lines
☒	☐	Palaeontology and archaeology
☐	☒	Animals and other organisms
☐	☒	Human research participants
☒	☐	Clinical data
☒	☐	Dual use research of concern

Methods

n/a	Involved in the study	
☒	☐	ChIP-seq
☒	☐	Flow cytometry
☒	☐	MRI-based neuroimaging

Animals and other organisms

Policy information about studies involving animals; ARRIVE guidelines recommended for reporting animal research

Laboratory animals	C57BL/6 (postnatal day 7) for single cell RNAseq and B6.CAST-Cdh23Ahl +/WT (5 females, 1 male, 11-28 weeks) for KLHDC7B quantitation.
Wild animals	No wild animals were used.
Field-collected samples	No field-collected samples were used.
Ethics oversight	All protocols were approved by the Institutional Animal Care and Use Committee in accordance with the Regeneron's Institutional Animal Care and Use Committee (IACUC).

Note that full information on the approval of the study protocol must also be provided in the manuscript.

Human research participants

Policy information about studies involving human research participants

Population characteristics	Participants were of European descent and the median age of individuals across all cohorts was between 58-76. Age, sex and ancestry principal components were included as covariates in all genetic association analyses.

Recruitment	Participants were recruited through health systems and biobanks. There can be some bias in the cohort based on ascertainment as individuals identified in hospital settings or by electronic health records may have more severe disease as opposed to individuals in a healthy population setting self-reporting the disease. In the health system based cohorts where cases and controls were defined based on the presence of hearing loss related ICD codes, there may be individuals with milder hearing loss that were not designated an appropriate ICD code and hence were labeled as controls.
Ethics oversight	All participants provided informed consent, and studies were approved by the individual Institutional Review Boards (IRBs) at the respective institutions. UK Biobank has approval from the North West Multi-Centre Research Ethics Committee (MREC; ref: 11/NW/0382), which covers the UK. It also sought the approval in England and Wales from the Patient Information Advisory Group (PIAG) for gaining access to information that would allow it to invite people to participate. The DiscovEHR study was approved by the Geisinger Health System Institutional Review Board. The BioMe Biobank is an ongoing research biorepository approved by the Icahn School of Medicine at Mount Sinai's IRB. The Ethical Committee at Lund University approved the Malmo Diet and Cancer Study (LU 51-90). The Finngen Biobank was approved by the Coordinating Ethics Committee of the Helsinki and Uusimaa Hospital District.

Note that full information on the approval of the study protocol must also be provided in the manuscript.
